# A multimodal cancer rehabilitation programme promoting sense of coherence for women treated for female reproductive cancers: a pilot randomised controlled trial

**DOI:** 10.1007/s11764-024-01630-2

**Published:** 2024-07-08

**Authors:** Ka Ming Chow, Carmen Wing Han Chan, Alexandra Leigh McCarthy, Jiemin Zhu, Kai Chow Choi, Ka Yi Siu, Alice Wai Yi Leung, Khanh Thi Nguyen

**Affiliations:** 1 The Nethersole School of Nursing, Faculty of Medicine, The Chinese University of Hong Kong, Hong Kong SAR, China; 2 Griffith Health, Griffith University, Brisbane, QLD Australia; 3 Department of Nursing, School of Medicine, Xiamen University, Xiamen, China; 4 Department of Obstetrics and Gynaecology, Prince of Wales Hospital, Hospital Authority, Hong Kong SAR, China

**Keywords:** Gynaecological cancer, Breast cancer, Rehabilitation, Diet, Distress, Physical activity, Sense of coherence, Quality of life

## Abstract

**Purpose:**

To investigate the feasibility, acceptability, and preliminary effects of a theory-driven multimodal cancer rehabilitation intervention (MCRI) programme among Hong Kong Chinese women treated for female reproductive cancers (FRC).

**Methods:**

A single-blinded randomised controlled trial was conducted in two regional hospitals in Hong Kong involving 35 women treated for FRC. The intervention group (*n* = 18) received a 12-week MCRI which included 30 modules of app-based health education and three nurse-led individual counselling sessions. The control group (*n* = 17) received attention from the research nurse through telephone calls. Sense of coherence, health-related quality of life, and cancer-specific distress were measured at baseline (T0), immediately after completion of the intervention (T1) and 12 weeks post-intervention (T2). Twelve intervention completers were interviewed to explore the acceptability of the programme.

**Results:**

Recruitment, consent, and retention rates, counselling session attendance rate, and app usage were satisfactory. The intervention participants reported to have significant improvement in physical well-being at T1 (Cohen’s *d* effect size (*d*) = 1.04, 95% CI 0.24, 1.83), sense of coherence (*d* = 0.76, 95% CI − 0.03, 1.54), and cancer-specific distress (*d* = 1.03, 95% CI − 1.83, − 0.21) at T2. Interviewed participants acknowledged the benefits of the programme and provided comments for improvement.

**Conclusions:**

The MCRI is found to be feasible and acceptable and may improve their sense of coherence, distress, and physical health. A full-scale trial using a larger and more representative sample is warranted to confirm the effects of the programme.

**Implications for Cancer Survivors:**

Women treated for FRC may be benefited from the MCRI in improving sense of coherence, physical well-being, and distress.

**Trial registration:**

This trial was registered on ISRCTN registry with ID ISRCTN73177277.

**Supplementary Information:**

The online version contains supplementary material available at 10.1007/s11764-024-01630-2.

## Introduction

Female reproductive cancers (FRC), which include breast, uterine, ovarian, cervical, vaginal, and vulvar cancers, accounted for nearly 40% of all cancers in women worldwide in 2020 [1]. Uterine, ovarian, cervical, vaginal, and vulval cancers are collectively named as gynaecological cancer. Although women with breast cancer and gynaecological cancer receive different treatment modalities, they all usually receive surgery plus adjunctive chemotherapy and radiotherapy. Hence, they report common treatment-induced problems typically faced by people treated for cancer such as fatigue, psychological distress, post-traumatic stress disorder, and anxiety [2, 3]. On top of those problems, they also suffer from specific cancer-related problems such as pain from lower limb lymphoedema in gynaecological cancer and upper limb lymphoedema in breast cancer, menopause-related symptoms, body image disturbance, and sexual dysfunction, as well as increased risk of developing chronic conditions such as obesity, osteoporosis, and diabetes, all of which affect their capacity to return to their normal roles and functions, resulting in a lower health-related quality of life (HRQoL) [2–5]. In view of the improved survival rates of women treated for FRC and its associated long-term impacts, there is an increasing demand for specialist cancer programmes that are customised to the specific needs of women being treated for FRC [6, 7].

Cancer rehabilitation programmes are designed to help patients manage symptoms and side effects and promote health and well-being after cancer treatment [8]. As information technology continues to advance, there is a growing body of evidence supporting the success of online cancer rehabilitation programmes for women after receiving cancer treatment [9–11]. For example, the Women’s Wellness After Cancer Programme (WWACP) was developed in Australia using an iBook and virtual health consultation to support women with blood, breast, and gynaecological cancer, aiming to promote and sustain healthy lifestyle behaviours [12]. This 12-week e-health intervention demonstrated improvement in general health, bodily pain, and physical and mental health for the target population [12]. Previously, our research team has culturally adapted the WWACP to the Hong Kong context, WWACPHK, using a four-stage approach proposed by Barrera and Castro [13], and the details are described in our prior work [14]. We pilot-tested the web-based version of WWACPHK on women treated for gynaecological cancer, demonstrating its feasibility and acceptability. The preliminary data indicated a large significant effect on enhancing exercise self-efficacy while the effects on psychological symptoms and HRQoL were small to medium and did not reach statistical significance [14].

The development of WWACP was underpinned by social cognitive theory, which emphasises on promoting self-efficacy for health behaviour change [15]. The self-efficacy concept encompasses the belief in individuals’ ability to control over their lives, including outcome expectancies and established goals [15]. However, the qualitative findings from our pilot study predominantly revolved around participants’ perception of the programme as a resource for coping with life after cancer [14]. Therefore, we have modified the WWACPHK based on a stress-resource-oriented theory—Antonovsky’s Salutogenic Model, which emphasises the concept of sense of coherence (SOC) [16]. SOC refers to an individual’s disposition to effectively cope with challenging experiences and to promote health and well-being. SOC integrates three key elements of the comprehensibility, manageability, and meaningfulness of a situation or disease [16]. The more an individual understands, manages, and finds meaning in a stressful condition, the better their ability to cope. It is evident that FRC with stronger SOC is correlated with lower levels of psychological issues (e.g. anxiety, depression, and stress) [17] and better HRQoL [18, 19]. Being the health professional who are most frequently in contact with the patients, nurses can play a key role in cancer rehabilitation programmes in which they act as a specific resistance resource to manage stressors and mobilise generalised resistance resources such as knowledge, beliefs, and farsighted coping strategies [20]. To strengthen SOC, we have added a nurse counselling component to the WWACPHK and adopted two effective strategies including empowerment and reflection during the nurse consultation sessions. Empowerment facilitates participants to mobilise their generalised resistance resources, while reflection helps to promote comprehensibility, manageability, and meaningfulness [21].

While existing evidence supports the effect of SOC promoting interventions among various populations such as older adults [22], people with schizophrenia [23], and women suffering from post-traumatic stress disorder [24], there is limited evidence of such interventions on cancer populations. To address this gap, we have developed a multimodal cancer rehabilitation intervention (MCRI) programme guided by the Salutogenic Model for Chinese women treated for FRC. This pilot study aims to test the acceptability and feasibility of this MCRI among Hong Kong Chinese women treated for FRC. The objectives are to (1) examine the feasibility of the trial design; (2) test the preliminary effects of the programme on SOC, cancer-specific distress, and HRQoL; and (3) collect views and comments on the programme for assessing acceptability.

## Methods

### Design

An assessor-blind, parallel pilot randomised controlled trial was conducted in the gynaecological or breast oncology clinics at two regional hospitals in Hong Kong. This study followed the CONSORT extension to pilot trials [25] and the CONSORT-EHEALTH checklist [26].

### Participants

Inclusion criteria for recruitment included women with primary diagnosis of FRC; within 3 months of completion of intensive cancer treatments (e.g. surgery, radiotherapy, and/or chemotherapy) but can be on maintenance therapies such as tamoxifen, trastuzumab, and bisphosphonates; over 18 years old; able to understand spoken Cantonese and to read Chinese; having internet-connected computing devices or smartphones; and consenting to participate. Individuals who had health conditions that could potentially affect their ability to understand information and complete questionnaires, such as visual impairment or pre-existing psychosis based on medical records and verification during the recruitment process, were excluded from the study. Based on the rule of thumb proposed by Browne [27] that the sample size for a pilot study should be at least 30 and the previous pilot study on the similar patient sample population [14, 28, 29], we aimed to recruit 40 participants, with 20 diagnosed with breast cancer and 20 diagnosed with gynaecological cancers.

### Randomisation, allocation concealment, and blinding

The enrolled participants were randomised in a 1:1 allocation ratio by an independent statistician using computer-generated random numbers. The sequentially numbered, opaque sealed envelopes were used to ensure allocation concealment. All data assessors, including those administering the instruments at follow-ups, were blinded. However, due to the nature of the intervention, it was not possible to blind the participants, the interviewer, and the nurse intervener.

### The intervention

Participants in the intervention group received the 12-week MCRI delivered by a research nurse. The MCRI is largely based on the web-based version of WWACPHK [14]. In the current study, we have incorporated the key concepts of the Salutogentic Model [20] (providing generalised and specific resistance resources to enhance comprehensibility, manageability, and meaningfulness) into WWACPHK, developed an mobile application (app) with interactive features, and added a nurse counselling component. In brief, the MCRI featured WWACPHK app as a generalised resistance resource and three individual telephone counselling sessions provided by the nurse as a specific resistance resource to enhance women’s SOC after cancer treatment. The intervention participants were given access to the WWACPHK app, which included 30 modules covering topics such as healthy diet, exercise, menopause-related symptoms and management, sleep, sexuality, body image, pelvic floor exercises, stress management, chronic disease prevention, and cancer screening. New information was released to the app daily for the first 3 weeks and then weekly for the remaining 9 weeks. Participants could also communicate with the research nurse through instant messaging within the app. The research nurse monitored participants’ activity on the app and provided reminder telephone calls if their accounts remained inactive for a week or more. Moreover, the research nurse conducted telephone counselling sessions with the participants in weeks 1, 6, and 12 which aimed to empower participants by instructing them on how to use the app, providing tailored health information, encouraging them to reflect on their beliefs, assumptions, knowledge, and goals to promote comprehensibility, manageability, and meaningfulness.

Participants allocated to the attention control group received basic information about the follow-up schedule during the baseline data collection. To minimise the effect of attention, participants in the control group also received attention from the research nurse during the same time period as the intervention group through telephone calls at weeks 1, 6, and 12. The nurse delivered general greetings and did not provide any specific interventions. After the completion of the study, participants in the control group received the eBook version of WWACPHK.

### Measurements

#### Feasibility

The recruitment rate (number of participants recruited per month), consent rate (number of consented participants divided by the number of eligible participants), retention rate (number of participants included in final analysis divided by the number of consented participants), counselling session attendance rate, percentage of modules accessed, and number of days active were recorded to assess feasibility.

#### Intervention outcomes

The Chinese version of the Sense of Coherence 13-item Scale (CSOC-13) was used to assess SOC, with items clustered in meaningfulness, manageability, and comprehensibility domains. Higher scores indicate higher SOC, and the scale has shown validity and consistency among Chinese women with cervical cancer [30].

Cancer-specific distress was measured by the 22-item Chinese version of the Impact of Events-Revised Scale (CIES-R), with items clustered in intrusion, avoidance, and hyperarousal domains. Higher scores indicate greater distress, and the scale has demonstrated good internal consistency [31].

The Hong Kong Chinese version of the MOS 36-item Short Form Health Survey version-2.0 (SF-36v2) was used to measure HRQoL, with items clustered into eight domains, including physical functioning, role physical, bodily pain, general health, vitality, social functioning, role emotional, and mental health. These eight domains can be further aggregated into physical health component and mental health component summary scores. Higher scores indicate better HRQoL, and the scale has shown good validity, consistency, and reliability among the adult population in Hong Kong [32].

#### Acceptability

To assess the participants’ acceptability of the programme, individual semi-structured interviews were conducted with participants in the intervention group via telephone calls to collect views and comments on the MCRI. All interviews were audio-recorded. A co-author conducted the interviews with reference to an interview guide (Supporting information S1).

#### Demographic and clinical characteristics

The demographic and clinical characteristics of the participants were collected using a data collection sheet which included age, education level, monthly household income, marital status, number of children, length of residence in Hong Kong, religious beliefs, type and stage of cancer, treatment modality for the disease, and time since treatment completion.

### Data collection

The research nurse approached eligible patients in the gynaecological or breast oncology clinics and explained the aims of the study. Information sheets about the study and consent forms were provided. Upon obtaining written consent, the patient’s demographic data were collected, and the instruments were administered during face-to-face interviews at baseline (T0) before random allocation. The instruments CSOC-13, CIES-R, and Hong Kong Chinese SF-36v2 were reassessed on completion of the programme (T1) and 12 weeks after completion (T2) by a blinded research assistant. Following programme completion, intervention participants were invited to participate in recorded telephone interviews to share their experiences and thoughts about the programme. To minimise potential experimenter influence, a co-author with rich experience in conducting qualitative interviews with cancer patients facilitated the telephone interviews with reference to the interview guide. Each interview lasted from 14 to 45 min. All participants did not receive any financial compensation for participation in the study.

### Data analysis

Statistical analyses were performed using SPSS version 28 (IBM Corp., Armonk, NY), and all tests were done using a two-sided 0.05 level of significance. Due to the challenge of assessing the normality of the continuous variables with the small sample size, non-parametric tests were adopted for all inferential analyses. Baseline characteristics between the intervention and control groups were compared using Mann–Whitney, chi-square, and Fisher’s exact test, as appropriate. The changes in outcomes at T1 and T2 with respect to T0 between the intervention and control groups were compared using the Mann–Whitney test and Cohen’s *d* effect size (95% confidence intervals). According to Cohen [33], effect sizes of 0.2 were considered small, 0.5 medium, and 0.8 large.

The interview recordings were transcribed verbatim, and the transcripts were analysed using NVivo 2020 (QSR International, USA). The thematic analysis approach by Braun and Clarke [34] was adopted to analyse the interview transcriptions. After reading one-third of the transcripts to become familiar with the data, two researchers (the first author and the interviewer, both involved in designing the study) independently identified initial codes from the early transcripts and then condensed similar codes into subthemes and themes through iterative discussions. This process involved continuous team comparisons and discussions to refine the subthemes and themes until reaching a consensus. Finally, the subthemes, themes, and selected quotations were translated by the interviewer fluent in both English and Chinese. The translations were then confirmed by the first author to ensure semantic equivalence.

### Rigour

Lincoln and Guba [35] outlined the four criteria for developing trustworthiness in qualitative research, including credibility, transferability, dependability, and confirmability. Transferability concerns about the generalisability of the findings to other settings or groups, which was not the aim of the qualitative study [36, 37]. To improve credibility, we recruited an independent researcher to conduct the interviews, provided examples of representative quotations, depicted the role and experience of the researchers, and had two researchers independently performed analysis and sought agreement on the final themes, subthemes, and codes [36]. To enhance dependability, the same interview guide (Supporting information S1) was used to guide all interviews [36]. Finally, to maximise confirmability, we provided a rich presentation of the quotes from the interviews organised under respective codes, subthemes, and overarching themes (Supporting information S2) [37].

## Results

### Feasibility

**Fig. 1:**
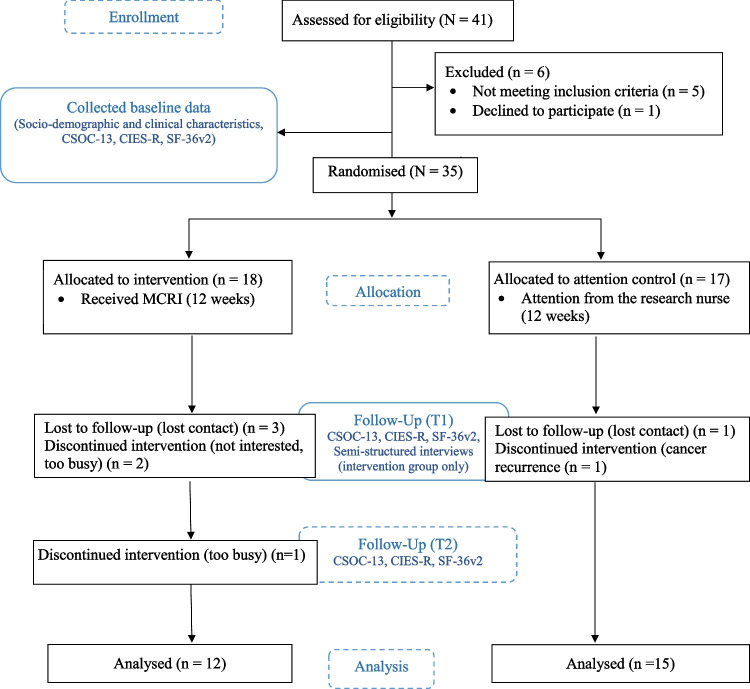
CONSORT flowchart of the study

Between October 2021 and January 2022, 35 out of 42 eligible participants (consent rate, 83.3%) were successfully recruited and randomised into either the intervention (*n* = 18) or control (*n* = 17) groups. As the recruitment period lasted for approximately 9 weeks, the recruitment rate was estimated to be 20 participants per month. Due to time limitations and the suspension of non-emergency outpatient services during the COVID-19 pandemic, the subject recruitment ended with a smaller sample size than originally planned. Nevertheless, a similar sample size has been adopted in our previous pilot study on a sample of women treated for gynaecological cancer [14]. At T1, seven participants dropped out, two of whom were not interested, three could not be contacted by phone, one experienced cancer recurrence, and one was too busy. At T2, one participant dropped out due to being too busy. The final sample included for analysis in this study was 27 resulting in a retention rate of 77.1% (see details in Fig. 1). Among the 18 participants who were randomised into the intervention group, all attended the first consultation session. However, five participants dropped out from the intervention before T1 having accessed less than seven app modules. Of the 18 intervention participants, the attendance rate for consultation sessions reached 87%, and on average, 64.6% of the 30 modules were viewed by the participants. The mean number of days active was 19.8 (SD = 16.1) days. Adopting the minimum dosage criteria of accessing more than 50% of the app modules and attending at least two consultation sessions as the threshold for intervention completion, the 13 participants who completed the T1 assessment were considered intervention completers. All participants did not report any adverse events during the study period.

### Demographics and clinical characteristics

Table 1 depicts the participants’ demographics and clinical characteristics. No significant differences were found in the participants’ demographics and clinical characteristics between the intervention and control groups at baseline. The mean age of the sample was 50.2 (SD = 6.6). The majority of the women were married and had at least one child (68.6%). Regarding clinical characteristics, almost 60% of the women had both breast cancer and stage one diagnosis. The majority received surgery plus radiotherapy and/or chemotherapy (74.3%).

**Table 1:** Baseline characteristics of the participants

Characteristics	Intervention (*n* = 18)		Control (*n* = 17)		*P*^a^
	Mdn or *N*	IQR or %	Mdn or *N*	IQR or %	
Age (years)	51.5	40.8, 56.0	51	47.5,53	0.78
Residence in Hong Kong (years)	44.5	32.5, 54.3	49	41.5,53	0.64
Education level					
Primary school or below	4	22.2%	3	17.6%	0.75
Secondary school	7	38.9%	9	52.9%	
University or above	7	38.9%	5	29.4%	
Monthly family income (Hong Kong dollars)					
< 10,000	3	16.7%	4	23.5%	0.46
10,000–29,999	5	27.8%	7	41.2%	
≥ 30,000	9	50%	4	23.5%	
No idea	1	5.6%	2	11.8%	
Marital status					
Married	10	55.6%	14	82.4%	0.15
Single/divorced/widowed	8	44.4%	3	17.6%	
Number of children					
0	7	38.9%	4	23.5%	0.81
1	5	27.8%	5	29.4%	
2	5	27.8%	7	41.2%	
3	1	5.6%	1	5.9%	
Religious belief					
No	10	55.6%	7	41.2%	0.51
Yes	8	44.4%	10	58.8%	
Cancer diagnosis					
Breast cancer	10	55.6%	10	58.8%	1.00
Gynaecological cancer	8	44.4%	7	41.2%	
Stage of cancer					
1	11	61.1%	9	52.9%	0.57
2	3	16.7%	5	29.4%	
3	4	22.2%	2	11.8%	
Do not know	0	0.0%	1	5.9%	
Types of treatment modalities received					
Surgery only	6	33.3%	3	17.6%	0.54
Surgery + chemotherapy	1	5.6%	2	11.8%	
Surgery + radiotherapy	4	22.2%	7	41.2%	
Surgery + chemotherapy + radiotherapy	7	38.9%	5	29.4%	

*Abbreviations*: *IQR* interquartile range, *Mdn* median

^a^Variables were compared between the two groups using Mann–Whitney, chi-square, and Fisher’s exact test, as appropriate

### Preliminary effects of the programme

Table 2 shows the comparison of the changes in intervention outcomes between the intervention and control groups. Overall, compared to the control group, the intervention group showed an improvement in most of the outcomes with small to large effect sizes at both T1 and T2. At T1, the intervention group experienced a significant greater improvement in the SF36v2-PCS score with a large effect size (*d* = 1.04, 95% CI 0.24, 1.83). At T2, the intervention group demonstrated a significant greater increase, with medium to large effect sizes, in CSOC-13 total score (*d* = 0.76, 95% CI − 0.03, 1.54) and manageability sub-scale score (*d* = 1.05, 95% CI 0.23, 1.86). Additionally, the intervention group demonstrated a significant greater reduction, with a large effect size, in CIES-R total score (*d* = 1.03, 95% CI − 1.83, − 0.21), intrusion sub-scale score (*d* = 0.91, 95% CI − 1.70, − 0.10), and hyperarousal sub-scale score (*d* = 0.91, 95% CI − 1.69, − 0.09).

**Table 2:** Summary of the changes in outcomes and effect size

Measures	T1-T0, Mdn (IQR)		ES (95% CI)	T2-T0, Mdn (IQR)		ES (95% CI)
	IG (*n* = 13)	CG (*n* = 15)		IG (*n* = 12)	CG (*n* = 15)	
CSOC-13						
Total score	2 (− 5.5, 14)	− 2 (− 7, 8)	0.33 (− 0.42, 1.08)	2.5(− 0.5, 6.5)	− 4 (− 7, 2)	0.76 (− 0.03, 1.54)*
Comprehensibility	0 (− 5, 8.5)	0 (− 2, 2)	0.26 (− 0.49, 1.01)	1 (− 1, 3)	0 (− 2, 1)	0.35 (− 0.42, 1.11)
Manageability	2 (− 0.5, 5)	− 1 (− 2, 2)	0.59 (− 0.18, 1.34)	2 (0, 5.3)	− 1 (− 3, 2)	1.05 (0.23, 1.86)*
Meaningfulness	1 (− 2.5, 3)	0 (− 3, 3)	0 (− 0.74, 0.74)	− 0.5 (− 2.8, 3.8)	− 2 (− 4, 0)	0.45 (− 0.32, 1.22)
CIES-R						
Total score	− 12 (− 21, − 2.5)	− 6 (− 9, − 3)	− 0.59 (− 1.35, 0.17)	− 12 (− 21, − 3)	− 3 (− 5, 0)	− 1.03 (− 1.83, − 0.21)*
Intrusion^^^	− 0.6 (0.8, 0.3)	− 0.5 (− 0.6, 0)	− 0.33 (− 1.08, 0.42)	− 0.5 (− 0.8, − 0.1)	− 0.1 (− 0.4, 0.3)	− 0.91 (− 1.70, − 0.10)*
Avoidance^^^	− 0.5 (− 1.3, 0)	− 0.1 (− 0.4, 0)	− 0.66 (− 1.42, 0.11)	− 0.5 (− 1.3, − 0.1)	− 0.1 (− 0.5, 0)	− 0.83 (− 1.61, − 0.03)
Hyperarousal^	− 0.3 (− 0.8, − 0.1)	− 0.2 (− 0.3, − 0.2)	− 0.37 (− 1.11, 0.39)	− 0.6 (− 0.7, − 0.1)	− 0.2 (− 0.3, 0.2)	− 0.91 (− 1.69, − 0.09)*
SF36v2						
PCS	5.7 (2.4, 11.9)	1.6 (− 2.0, 6.0)	1.04 (0.24, 1.83)*	2.7 (− 1.9, 13.0)	− 0.7 (− 3.3, 3.0)	0.71 (− 0.08, 1.48)
MCS	5.5 (− 4.3,7.5)	2.1 (0.5, 8.8)	− 0.07 (− 0.81, 0.68)	3.0 (− 3.0, 10.5)	2.8 (0.7, 8.0)	− 0.02 (− 0.78, 0.74)

*Abbreviations*: *CSOC-13* Chinese version of the Sense of Coherence 13-item Scale, *CIES-R* Chinese version of Impact of Events-Revised Scale, *SF-36v2* the MOS 36-item Short Form version 2, *PCS* physical component summary, *MCS* mental component summary, *T0* baseline, *T1* on the completion of the programme, *T2* 12 weeks after completion, *IG* intervention group, *IC* control group, *IQR* interquartile range, *Mdn* median, *ES* Cohen’s *d* effect size (positive ES favours intervention and vice versa), *CI* confidence interval

^*^p < 0.05

^*^*^Mean of all items in the subscale

### Acceptability of the intervention

All intervention completers (*n* = 13) were recruited for individual interviews via phone to express their perceptions and opinions about the programme. However, one refused to participate due to busy schedules. The qualitative data from the 12 intervention completers generated two themes, including the perceived benefits of the programme and other comments about the programme. Supporting information S2 summarises the themes, subthemes, codes, and sample quotes.

#### Theme 1: perceived benefits of the WWACPHK

All interviewed participants expressed a positive impression of the programme and reported experiencing beneficial outcomes after the intervention. Specifically, they appreciated the information and psychological support provided in the programme, which facilitated positive lifestyle changes.

##### Subtheme 1.1: information support

Most participants found the information provided in the mobile app useful and comprehensive. In particular, a few participants commented that they trusted the accuracy and credibility of the information provided. Among the various topics, most participants found the diet-related information, particularly healthy food choices and fruit and vegetable intake, to be relevant and useful. Following the COVID-19 outbreak, some participants reported a decrease in physical activity. The physical activity information reminded them of the importance of staying active despite the restrictions posed by the pandemic. Other than diet and physical activity, a few women reported increased awareness about health screening and body checks.

In addition to the information provided in the app, some participants valued tailored information provided by the research nurse to help them cope with the treatment effects at post-treatment, which relieved their worries.

I wasn’t feeling well for a few days. It was like having menstrual pain. I shared this with the nurse. After she explained to me, I felt relieved—Participant 4.

##### Subtheme 1.2: psychological support

Throughout the study period, a majority of the participants experienced negative emotions associated with various aspects, including fear of cancer recurrence, uncertainty about the future, concerns about body image, family burden, fertility issues, and work-related stress. They appreciated the opportunity to share their concerns and worries with the research nurse, which they were hesitant to share with their friends and family.

It’s really good to have someone to talk to. I feel like I can have a deeper conversation with this person [the nurse], and she has more [professional] knowledge. It’s different from talking to my friends.—Participant 6.

##### Subtheme 1.3: positive lifestyle changes

After reading the information about healthy lifestyles in the app, the majority of participants reported initiating dietary changes, which included drinking more water, consuming more fruit and vegetables, reducing meat consumption, limiting unhealthy snacks, and developing a habit of eating breakfast. On the other hand, almost half of the participants either maintained their exercise habits or initiated a new exercise routine. Those who did not have an exercise routine identified barriers such as the side effects of treatments, restrictions posed by the pandemic, or being too busy.

For example… I now pay more attention to vegetables and fruits.—Participant 8.

#### Theme 2: other comments about the programme

Participants were asked to provide comments about the intervention and any difficulties they had encountered during the intervention. Their comments are summarised below.

##### Subtheme 2.1: dosage of intervention

Most participants commented that the daily release of new reading materials during the first 3 weeks was appropriate. However, some forgot to read the information in the app from week four to week 12 when the frequency of updates was reduced to once a week.

##### Subtheme 2.2: technical issues

Participants reported encountering technical issues when using the app. The most commonly reported technical issue was auto-logout, followed by poor layout of record forms, low video quality, and non-functional hyperlinks. They suggested addressing these issues to enhance the user-friendliness of the app.

I noticed that there are a lot of record forms, and they are quite complicated—Participant 10.

##### Subtheme 2.3: “Chat With Nurse” function

Only one participant had tried using the “Chat With Nurse” function in the app. She was satisfied with the nurse’s follow-up call after leaving a message. Nevertheless, the majority of participants agreed that this messaging function was convenient for asking questions, as they could leave a message for the nurse and receive follow-up at a later time.

This kind of support is like… you can mark down any questions you have, and you can send out your questions anytime. I think it is good.—Participant 6.

##### Subtheme 2.4: format of nurse counselling session

Although the majority of participants expressed a preference for telephone consultations, one participant expressed a preference for video consultations. That participant believed that the nurse could provide better care through observing the non-verbal cues in video consultations.

I think that phone consultation is more acceptable. Sometimes, it’s not possible to take video calls in public places. Video calls can only be used in specific locations.—Participant 7.

## Discussion

This study represents the first endeavour to assess the feasibility and acceptability of a theory-driven MCRI to promote SOC and HRQoL for women treated for FRC in Chinese settings. In this pilot RCT, we found that the MCRI is feasible and acceptable to our target population and demonstrated potential benefits on their SOC, cancer-specific distress, and physical well-being. We also collected suggestions for improvement for the development of a future larger-scale RCT.

The feasibility of the MCRI was supported by high consent rate (88.3%), consultation attendance rate (87%), satisfactory retention (77.1%), and app usage (65.6% access rate). The subject retention rate in this study was slightly lower than that reported in our previous web-based pilot study [14] but still higher than other mHealth-based interventions [38]. Although some technical issues were reported, feedback from the participants confirmed the programme’s acceptability, with positive remarks during interviews expressing gratitude for the programme’s informational and psychological support, leading to beneficial lifestyle changes. Specifically, intervention participants found the app’s information comprehensive and useful, particularly regarding dietary choices and physical activity, and they appreciated the personalised support from the research nurse that effectively helped them manage treatment side effects and emotional concerns. This finding aligns with that reported in the Lifestyle and Empowerment Techniques in Survivorship of Gynaecologic Oncology (LETSGO) study in Norway, in which women treated for gynaecological cancer received two nurse-led consultations, along with the health education materials provided in the LETSGO app for 6 months [39]. In that study, participants perceived the nurse consultations appropriate and useful and appreciated the opportunity to discuss physical, psychological, and lifestyle concerns with the nurse [39]. Likewise, the important role of nurses in providing person-centred support to help cancer patients manage treatment side effects and emotional concerns has been acknowledged in previous studies [40, 41].

Despite a small sample size, this study demonstrated that the intervention effectively enhanced the physical well-being with a large effect size immediately after the completion of the intervention and sustained positive effects with medium to large effect sizes on SOC and cancer-specific distress at 12 weeks post-intervention. The positive effect on physical well-being as measured by SF36v2 aligns with the findings of the full-scale WWACP [12]. As WWACP is a multidimensional lifestyle intervention that promotes holistic health behaviours, our study provides additional evidence to support the effects of such interventions on physical well-being of women treated for FRC.

Regarding SOC, we observed an improvement in the manageability aspects as well as overall SOC in the intervention group, as compared to the control group, at both follow-ups with a further increase in effect size at the later follow-up. These findings suggest that the effects of the MCRI on SOC extend beyond the intervention period and are sustained at 12 weeks after completion of the programme. Experiential learning, empowerment, knowledge transfer, and psychological support from the nurse may have contributed to the improvement in SOC [21, 42]. The continued post-intervention improvement indicates that it takes time for them to apply the resistance resources acquired in this programme to effectively cope with stressors. In view of the small and non-significant effects on the comprehensibility and meaningfulness aspects of SOC, more efforts should be made during the nurse consultation sessions to facilitate patients’ reflection on their understanding and foster a sense of purpose in their survivorship journey.

Similar to SOC, there was a reduction in cancer-specific distress in the intervention group, as compared to the control group, at both follow-ups with a further increase in effect size at later follow-up. This finding suggests that promoting SOC may be an effective strategy to alleviate cancer-specific distress. We posit that individuals with stronger SOC are inclined to use more coping strategies and select more appropriate strategies to cope with specific stressors [43]. In contrast, women with weak SOC tend to employ fewer coping strategies, use more emotional expression, and seek social and spiritual support, but less frequently use acceptance as a coping mechanism [44]. Acceptance in the context of cancer involves patients acknowledging and embracing their life with the illness without avoidance, denial, or judgment, accepting the losses caused by the illness, finding meaning in the experience, and endeavouring to control it [45, 46]. Thus, a strong SOC patient is more likely to have higher levels of acceptance, which can lead to reduced distress. Alternatively, qualitative findings revealed that intervention participants had the opportunity to share their concerns with nurses, which may have elicited a sense of relief and support thereby leading to a reduction in distress.

Finally, some intervention participants encountered technical issues while using the app. These issues should be addressed in the full-scale study to improve user experience and maximise the intervention effects. If the positive effects in our study are demonstrated in a larger RCT, this intervention can be incorporated into routine cancer survivorship care, contributing to improving the overall quality of nursing care.

### Limitation

A key strength of our study was that we incorporated measures to establish the trustworthiness criteria outlined by Lincoln and Guba [35] in the qualitative study. However, it is important to acknowledge several limitations. Firstly, due to the impact of COVID-19 and time constraints, the pilot study was conducted with a smaller sample size than planned. Given the underpowered sample size, the quantitative results should be interpreted with caution. A full-scale RCT using a larger and more representative sample is necessary to verify the effects of the MCRI on the outcomes. Secondly, while assessors were blinded, the participants and the interveners were not, potentially leading to bias in the study findings. Third, intervention completers reported positive changes in dietary and physical activity behaviours after participation; however, validated questionnaires were not used to examine these changes. Further studies should adopt validated questionnaires to verify the effects of the intervention on health behaviour changes. Finally, given that motivation is a key factor promoting lifestyle changes in cancer patients [40], the positive lifestyle changes reported in intervention completers might be subject to selection bias of enrolling only highly motivated patients in our study. It is possible that patients with lower motivation levels were underrepresented in our sample.

## Conclusion

Implementing the MCRI is feasible and acceptable among Chinese women treated for FRC. Our study demonstrated significant improvements in SOC, cancer-specific distress, and physical well-being after participating in the MCRI among this cohort. Intervention participants acknowledged the benefits of the programme and provided comments for improvement. Considering the positive findings from this pilot study, a full-scale RCT will be conducted to confirm the effects of the MCRI.

## Supplementary Information

Below is the link to the electronic supplementary material.

Supplementary file1 (DOCX 17 KB)

Supplementary file2 (DOCX 30 KB)

## Data Availability

The anonymous dataset will be available from the corresponding author on reasonable request.
