# Supplementary material for: A multimodal cancer rehabilitation programme promoting sense of coherence for women treated for female reproductive cancers: a pilot randomised controlled trial
Source: J Cancer Surviv. 2024 Jul 8;20(1):28–38. doi: 10.1007/s11764-024-01630-2 (PMC12906564; doi:10.1007/s11764-024-01630-2)
Supplement: Supplementary file 1 — Supplementary file1 (DOCX 17 KB) [file 11764_2024_1630_MOESM1_ESM.docx]

**Supporting information S1. Interview guide**

1. What is your general impression of the multimodal cancer rehabilitation intervention (MRCI) programme?
2. Tell me about your experience of this programme.
3. How did the interventions help you understand various stressors?
4. How did the interventions help you identify available resources for managing stress in your environment?
5. How did the interventions help you manage stress?
6. How did the interventions help you in your daily life?
7. Describe for me how you modify your lifestyle (daily life) as a result of this programme.
8. Describe for me how you set goals when dealing with stressors as a result of this programme.
9. How did you apply the information from the programme in your daily life?

Probe for dietary management, physical activity, pelvic floor exercises, sleep measures and quality, stress management, menopausal symptoms, sexuality, body image and cancer screening behaviour.

1. Do you have any comments or recommendations on the programme, including the content, mode of delivery, frequency, duration, intervener?
2. Do you recommend the programme to others having a similar situation?
