# Supplementary material for: A multimodal cancer rehabilitation programme promoting sense of coherence for women treated for female reproductive cancers: a pilot randomised controlled trial
Source: J Cancer Surviv. 2024 Jul 8;20(1):28–38. doi: 10.1007/s11764-024-01630-2 (PMC12906564; doi:10.1007/s11764-024-01630-2)
Supplement: Supplementary file 2 — Supplementary file2 (DOCX 30 KB) [file 11764_2024_1630_MOESM2_ESM.docx]

**Supporting information S2. Themes, subthemes, codes and sample quotes.**

| Theme | Subtheme | Code | Sample quote |
| --- | --- | --- | --- |
| Perceived benefits of the programme | Information support | Diet-related information | ‘For me, the diet-related information is useful, especially the recommended healthy foods. I can check for myself what kind of food I can add to my current diet. If there are some foods that I haven’t eaten before, I should try to make up for it.’ *(P5)*  ‘Indeed, it has a lot of information. It’s good for a patient to read. When you read more of it, it becomes ingrained in your mind. Isn't that right? Sometimes, I chitchat to my colleagues at work and share with them the importance of eating more vegetables for a balanced diet.’ *(P8)* |
|  |  | Exercise-related information | ‘I have learnt, for example, what should be done [to improve my health], like exercising, avoiding certain foods, and so on, that can make a difference. I gained some new information. It's quite comprehensive and I don't have to search for each specific topic online by myself.’ *(P4)*  ‘It’s quite helpful, especially the exercise-related information. Because of the current pandemic situation, I have been exercising less than before. But when I read the information in the app, it reminds me to try and find time to do more exercise.’ *(P12)* |
|  |  | Health check | ‘Yes, I am more aware of health issues now. When I have time, I want to book an appointment for health check.’ *(P6)*  ‘I now realise the importance of taking care of our bodies. In the past, I didn't do regular body checks, like checking bone density or getting a chest X-ray. But now I will go for those check-ups.’ (P12) |
|  |  | Tailored information provided by nurse | ‘Okay... For example, during the Lunar New Year, I wasn't feeling well for a few days. It was like having menstrual pain. I shared this with the nurse. After she explained to me, I felt relieved.’ *(P4)*  ‘The nurses are professionals. The whole programme is designed in such a way that if you need someone to talk to or if you have any questions, you can actually [ask a nurse] ... When we go for follow-up appointments, there is limited time for questions. However, [with this app,] if there's any sudden issues, we can reach out to a nurse. I remember that if you type your questions [in the app], a nurse will follow up very soon.’ *(P9)* |
|  | Psychological support | NA | ‘It's really good to have someone to talk to. I feel like I can have a deeper conversation with this person [the nurse], and she has more [professional] knowledge. It's different from talking to my friends... There's a significant difference here. Reaching out to professionals, like nurses, allows me to share more and ask questions. This is totally different from just sharing with friends.’ *(P6)*  ‘Yes, she [the nurse] is really great. When I'm feeling really down, but I don't know... why I felt this way even though I've finished all the treatments. Maybe it's just... you know, the recovery process. I have to go back to work, but I don't know how to move forward. I feel helpless, you know. I don't know what to do... I've been feeling really bad lately. The nurse told me that if I feel really down and need someone to talk to, I can reach out to her. After talking to her, I actually felt much better.’ *(P7)* |
|  | Positive lifestyle changes | Dietary changes | ‘For example... I now pay more attention to vegetables and fruits. In the past, when I ordered take away, I didn't order vegetables often. But now, I always include some vegetables in my orders, like boiled dishes without oyster sauce. As for fruits, before, I just ate oranges. But now... I intentionally buy apples, grapes, and have increased the overall fruit consumption.’ *(P8)*  ‘I have eaten more fruits and vegetables. Yeah, even if I really crave meat, I still try to balance it out. I tell myself... if you want to eat chicken, then have three bites of vegetables first. I have these kind conversations with myself. Before, I wouldn't choose [vegetables], but now I consciously try to eat more… Now I eat more vegetables than meat.’ *(P9)*  ‘In the past, I didn't eat breakfast, but now I started eating breakfast.’*(P12)* |
|  |  | Exercise habit changes | ‘I have tried the stretching exercises [in the app]. Because I am quite heavy, about 200 pounds… There are some movements that I must adjust. But I do try to keep doing exercise, like playing badminton and swimming.’ *(P9)*  ‘Indeed, I am now doing the exercises that I used to do before having cancer, for example, a 30-minute walk. It’s my habit.’ *(P12)* |
|  |  | Barriers to exercise | ‘I haven't really been doing any exercise lately. When I first tried these exercises, I found them too intense for my body. So, I stopped for a while, and since then, I haven't tried again. But I do know that exercising is beneficial, especially for recovery. If I gradually start doing it again and keep doing it, I'm sure it will be helpful.’ *(P2)*  ‘I don’t have time [to exercise] because I have night shift. I usually sleep and rest during the day.’ *(P11)* |
| Other comments about the programme | Dosage of the intervention | Appropriate frequency | ‘It’s appropriate. If it’s too intense, some things might not have happened yet, right? I feel like the timing is okay... so as the frequency. I don’t think it needs to be too intense.’ *(P4)*  ‘I think it’s appropriate because you need to digest the content. It takes some time. Around a week in the later stage is okay.’ *(P11)* |
|  |  | Forgot to login | ‘For me, I have used this app quite often, especially in the beginning. However, as it started updating only once a week, I used it less often.’ *(P7)*  ‘I used to check it every day and it became a habit. However, it updated less frequently after a while. Then I always forgot to check because of my busy work schedule.’ *(P8)* |
|  | Technical issues | Auto-logout | ‘Once I got logged out automatically, I need to figure out how to login again. In the beginning, I didn't save the password on my phone, so I always forget it and have to search for the paper that I wrote it down.’ *(P3)* |
|  |  | Poor layout of record forms | ‘I noticed that there are a lot of record forms, and they are quite complicated. Initially, I tried to fill in the forms, but the number of cells, especially in the weekly records, was too overwhelming. I felt very stressed when I completed the forms. I know you want to collect more data, but it might make the patients feel uncomfortable. Maybe in the future, if you could have one page for each day, it would be easier to handle. Having too many cells on one page just makes it hard, and we might need to zoom in to read the words.’ *(P10)*  ‘I can write down my feelings, but I hope there is some feedback. In that way, I am not just talking to the air. Hmm, I think it's better this way… a little bit of interaction, not too much.’ *(P8)* |
|  |  | Low video quality | ‘The videos are on YouTube. I mean the exercise demonstration videos. I can only watch them on my phone, but I can't display them on TV. I want to follow the videos, but it's difficult because the screen is too small.’*(P7)* |
|  |  | Hyperlink not working | ‘There are so many hyperlinks, but they are not working. I think I can’t manage to type out the long URLs. But if those links are functional, I think it would be even more helpful.’ *(P8)* |
|  | “Chat with nurse” function | NA | ‘For those who need support, I think this function is convenient. Using WhatsApp could bother the nurses in their personal time. This app seems like a better choice.’ *(P4)*  ‘This kind of support is like… you can mark down any questions you have, and you can send out your questions anytime. I think it is good.’ *(P6)* |
|  | Format of nurse counselling session | Telephone-based | ‘I think that phone consultation is more acceptable. Sometimes, it's not possible to take video calls in public places. Video calls can only be used in specific locations.’ *(P7)*  ‘I think phone consultation is more convenient.’*(P8)* |
|  |  | Video-based | ‘I think video chats are more interesting! It’s important that you let the participants choose what works best for them and what makes them feel comfortable. In my opinion, video calls can enhance understanding and allow for more detailed explanations. Besides, as a professional nurse, I think you can pick up on any non-verbal cues during a video call.’ *(P9)* |
